# Prevalence of genetic risk factors related with thrombophilia and hypofibrinolysis in patients with osteonecrosis of the femoral head in Poland

**DOI:** 10.1186/1471-2474-14-264

**Published:** 2013-09-11

**Authors:** Jacek Gagala, Monika Buraczynska, Tomasz Mazurkiewicz, Andrzej Ksiazek

**Affiliations:** 1 Orthopaedic and Traumatology Department, Medical University of Lublin, ul. Dr K Jaczewskiego 8, 20-954 Lublin, Poland; 2 Department of Nephrology, Medical University of Lublin, ul. Dr K Jaczewskiego 8, 20-954 Lublin, Poland

**Keywords:** Osteonecrosis, Femoral head, Factor V Leiden, Prothrombin, MTHFR, TPA, Polymorphism

## Abstract

**Background:**

The etiology of osteonecrosis of femoral head (ONFH) has not been fully elucidated. Increased intravascular coagulation and/or hypofibrinolysis have been proposed as pathogenic mechanisms. Previous reports demonstrated significant association between incidence of ONFH and polymorphisms of genes related with thrombophilia especially in Caucasian subjects. The aim of our study was to evaluate the relationship between genetic mutations leading to coagulation disorders and ONFH in Polish patients.

**Methods:**

We have investigated the frequencies of four markers among 68 unrelated individuals with clinically and radiographically documented ONFH and among 100 healthy unrelated blood donors in Eastern part of Poland. The three genes were involved in thrombophilia: factor V Leiden (G1691A), prothrombin (G20210A), Methylenetetrahydrofolate Reductase (MTHFR C677T) and one in hypofibrinolysis: Tissue Plasminogen Activator (PLAT TPA25 I/D). The samples were genotyped with polymerase chain reaction followed by restriction enzyme analysis for the restriction fragment length polymorphisms. The allele and genotype frequencies were analyzed in the relation to ONFH etiology (idiopathic and secondary), gender, age (patients younger or older than 50 years) and the number of affected joints (unilateral or bilateral ONFH).

**Results:**

No significant difference in allele frequencies between patients and control groups were observed in genes involved in thrombophilia. We have found a statistically significant increased frequency of D allele of PLAT TPA 25 I/D polymorphism between the entire group of patients with ONFH and controls (p=0,026, OR=1,54, CI 0,99-2,4). D allele frequency was also significantly increased in patients with primary ONFH (p=0,009, OR=1,81 CI 1,1-3,01), in males (p= 0,013; OR 1,74; 95% CIs 1,08-2,78), patients older than 50 years (p= 0,018, OR= 2,04; 95% CIs 1,09-3,82) and in cases with bilateral ONFH (p= 0,01; OR= 1,92; 95% CIs 1,13-3,27) (Table 9). The differences in DD homozygous genotype frequency were statistically significant for patients with idiopathic ONFH compared with control group (p=0,023, OR=2,75, CI 0,99-7,9) and in cases of bilateral ONFH (p=0,034; OR 3,12; 95% CIs 1,06-9,18) (Table 10). The frequencies of ID heterozygous genotype were statistically significantly higher in entire group of patients with ONFH (p=0,004 OR 2,71; 95% CIs 1,32-5,57), idiopathic ONFH (p= 0,01; OR 2,91; 95% CIs 1,24-6,87), males (p=0,0007; OR 3,75; 95% CIs 1,67-8,42), patients older than 50 years (p=0,001; OR 6,89; 95% CIs 1,87-25,84) and in cases with bilateral ONFH (p=0,009; OR 3,19; 95% CIs 1,26-8,03).

**Conclusion:**

The results suggest that inherited hypofibrinolysis is a risk factor of idiopathic ONFH in Polish population.

## Background

Osteonecrosis (ON) or avascular necrosis (AVN) is a clinical entity characterized by death of bone cellular components due to interruption of blood supply [1]. The actual prevalence of ONFH is unknown. The estimated 10 000 to 20 000 new cases of ONFH are diagnosed every year in the United States [2]. ONFH occurs mainly in young people. The evolution of the disease leads to fracture of subchondral bone, collapse of the femoral head with subsequent degenerative changes of hip joint [3]. ONFH has traditionally been classified as idiopathic or secondary, depending on the absence or presence of known causes [1]. The most common risk factors of secondary ONFH include: the use of corticosteroids, alcoholism, hyperlipidemia, lipid storage diseases, hyperbaric exposure, pregnancy, SLE, solid organ transplantations and chemotherapy. The etiology and pathophysiology of ONFH have not been fully elucidated. An Increased tendency of intravascular coagulation has been proposed as pathogenic mechanism of ONFH in adults at the beginning of 90s [4]. Both thrombophilia (increased tendency to form thrombi) or/and hypofibrinolysis (reduced ability to lyse thrombi) may be the causes of ONFH in adults [5-7]. The same thrombophilia/hypofibrinolysis pathomechanism has been reported in ONFH in children (Legg-Calvé-Perthes disease) [8,9].

The aim of this study was to evaluate the relationship between genetic mutations leading to coagulation disorders and ONFH in Polish patients.

## Methods

### Patients

A group of 68 unrelated adult individuals were involved in this study. All the patients were Caucasians of Polish origin coming from Eastern part of Poland. Patients were admitted to the Department of Orthopaedics and Traumatology for operative treatment of ONFH. ONFH was diagnosed with clinical examination and radiographic analysis. Patients without characteristic risk factors related with ON were classified as idiopathic ONFH (n=45). The cause of ONFH was established in 23 patients: the use of corticosteroids (n=11), chemotherapy (n=7), alcoholism (n=4), renal transplantation (n=1). These patients were classified as secondary ONFH. Patients with idiopathic and secondary ONFH did not differ in the relation of average age and gender distribution. Ficat and Arlet classification [10] was used for radiographic evaluation. AP and axial view x-rays of both hips were taken in all of the patients. MRI was performed to confirm the diagnosis of ONFH in patients without x-ray changes. ONFH was present in one hip in 29 patients and in both hips in 39 patients. Stage I according to Ficat and Arlet was seen in 11 hips, stage II in 19, III in 24 and IV degree in 53 hips. Healthy control subjects (n=100) without any clinical signs and family history of ONFH were recruited among blood donors and hospital staff. All the participants in the control group also came from Eastern part of Poland. Control group consisted of 20 women and 80 men with average age of 36,6 (range from 20 to 55) years.

A written informed consent for genetic studies was obtained from all the patients and subjects from the control group. The study protocol was evaluated and approved by the Ethics Committee at the Medical University in Lublin.

### Genetic testing

Genomic DNA was isolated from peripheral blood leukocytes using the method described by Madisen et al. [11] with minor modifications. The samples were genotyped using the polymerase chain reaction under conditions previously described by authors [12-15] listed in Table 1.

**Table 1:** Panel of markers included in the study

**Gene**	**Polymorphism**	**Type**	**References**
Factor V Leiden	G1691A	RFLP	Zoller et al. [12]
Prothrombin	G20210A	RFLP	Poort et al. [13]
MTHFR	C677T	RFLP	Friedman et al. [14]
PLAT TPA 25	I/D	Ins.Alu	Tishkoff et al. [15]

### Statistical analysis

All the calculations were performed using SPSS for Windows 17.0. Normally distributed data are presented as means ± SD. Genotype distribution and allele frequencies were compared between groups using x^2^ test and Fisher’s exact test, respectively. Odds ratios (ORs) and 95% confidence intervals (CIs) were calculated using relevant 2×2 contingency tables.

## Results

We genotyped the polymorphisms of four genes related to coagulation abnormalities in 68 patients with ONFH and 100 healthy controls. Clinical characteristics of the study group are summarized in Table 2. There were no significant differences between the groups with respect to age and gender ratio, the number of affected joints and risk factors. We have found high incidence of smokers in idiopathic ONFH group. We could not reveal that smoking is a risk factor of ONFH because of lack of data on smoking in the control group. Allele and genotype frequencies of ONFH patients are presented in Tables 3 and 4. The genotype frequencies of the four polymorphisms analyzed were in Hardy Weinberg equilibrium. We analyzed the allele and genotype frequencies in the relation to ONFH etiology (idiopathic and secondary) (Tables 3 and 4), gender (Tables 5 and 6), age (patients younger or older than 50 years) (Tables 7 and 8) and the number of affected joints (unilateral or bilateral ONFH) (Tables 9 and 10). The genotype and allele frequencies did not differ for Factor V Leiden, prothrombin and MTHFR gene polymorphisms for patients with ONFH in comparison to controls. We have found a statistically significant increased frequency of D allele of PLAT TPA 25 I/D polymorphism between the entire group of patients with ONFH and controls (p=0,026, OR=1,54 ,CI 0,99-2,4) (Table 3). D allele frequency was also significantly increased in patients with primary ONFH (p=0,009, OR=1,81 CI 1,1-3,01) (Table 3), in males (p= 0,013; OR 1,74; 95% CIs 1,08-2,78) (Table 5), patients older than 50 years (p= 0,018, OR= 2,04; 95% CIs 1,09-3,82) (Table 7) and in cases with bilateral ONFH (p= 0,01; OR= 1,92; 95% CIs 1,13-3,27) (Table 9). The differences in DD homozygous genotype frequency were statistically significant for patients with idiopathic ONFH compared with control group (p=0,023, OR=2,75, CI 0,99-7,9) (Table 4) and in cases of bilateral ONFH (p=0,034; OR 3,12; 95% CIs 1,06-9,18) (Table 10). The frequencies of ID heterozygous genotype were statistically significantly higher in entire group of patients with ONFH (p=0,004 OR 2,71; 95% CIs 1,32-5,57) (Table 4), idiopathic ONFH (p= 0,01; OR 2,91; 95% CIs 1,24-6,87) (Table 4), males (p=0,0007; OR 3,75; 95% CIs 1,67-8,42) (Table 6), patients older than 50 years (p=0,001; OR 6,89; 95% CIs 1,87-25,84) (Table 8) and in cases with bilateral ONFH (p=0,009; OR 3,19; 95% CIs 1,26-8,03) (Table 10).

**Table 2:** Characteristics of ONFH study group

	**Osteonecrosis N=68**	**Idiopathic osteonecrosis n=45**	**Secondary osteonecrosis n=23**
Gender (men/women)	56/12 (82,35%/17,65%)	38/7 (84,44%/15,56%)	18/5 (78,26%/21,74%)
Age (years)	44,98 (15-77)	44,42 (15-77)	46,08 (20-74)
Bilateral ONFH	39 (57,35%)	23 (51,11%)	16 (69,56%)
Smoking	27 (39,7%)	21 (46,66%)	6 (26,06%)
Diabetes mellitus	8 (11,76%)	4 (8,88%)	4 (17,39%)
Coronary disease	3 (4,41%)	2 (4,44%)	1 (4,34%)
Thrombosis	1 (1,47%)	0	1 (4,34%)
Leriche syndrome	1 (1,47%)	1 (2,22%)	0
Hypertension	13 (19,11%)	8 (17,77%)	5 (21,73%)
Hyperlipidemia	1 (1,47%)	1 (2,22%)	0

**Table 3:** Allele frequencies of four polymorphisms in ONFH patients (total, idiopathic and secondary) and controls

**Allele**	**Controls N=200**	**Patients**		
		**Total N=136**	**Idiopathic n=90**	**Secondary n=46**
Factor V Leiden				
A	0,03	0,0225	0,0333	0
G	0,97	0,9775	0,9667	1,0
Prothrombin				
A	0,01	0,0074	0	0,0218
G	0,99	0,9926	1,0	0,9782
MTHFR				
T	0,28	0,3161	0,3444	0,2608
C	0,72	0,6839	0,6556	0,7392
TPA				
D	0,365	0,4705^a^	0,5111^b^	0,3478
I	0,6350	0,5295	0,4889	0,6522

^a^p =0,026; odds ratio (OR) 1,54; 95% confidence intervals (CIs) 0,99-2,4.

^b^p=0,009; OR 1,81; 95% CIs 1,1-3,01.

**Table 4:** Genotype frequencies of four polymorphisms in ONFH patients (total, idiopathic and secondary) and controls

**Genotype**	**Controls N=100**	**Patients**		
		**Total N=68**	**Idiopathic n=45**	**Secondary n=23**
Factor V Leiden				
GA	3(3%)	3(4,41%)	3(6,67%)	0
GG	97(97%)	65(95,59%)	42(93,33%)	23(100%)
Prothrombin				
GA	2(2%)	1(1,47%)	0	1(4,35%)
GG	98(98%)	67(98,53%)	45(100%)	22(95,65%)
MTHFR				
TT	7(7%)	4(5,88%)	4(8,89%)	0
CT	42(42%)	35(51,47%)	23(51,11%)	12(52,17%)
CC	51(51%)	29(42,65%)	18(40%)	11(47,83%)
TPA				
DD	18(18%)	13(19,12%)	11(24,44%)^a^	2(8,7%)
ID	37(37%)	38(55,88%)^b^	24(53,33%)^c^	14(60,87%)
II	45(45%)	17(25%)	10(22,22%)	7(30,43%)

^a^ p=0,023; odds ratio (OR) 2,75; 95% confidence intervals (CIs) 0,99-7,95.

^b^p=0,004 OR 2,71; 95% CIs 1,32-5,57.

^c^p= 0,01; OR 2,91; 95% CIs 1,24-6,87.

**Table 5:** Allele frequencies of four polymorphisms in ONFH patients according to gender (total, female and male) and controls

**Allele**	**Controls N=200**	**Patients**		
		**Total N=136**	**Female n=24**	**Male n=112**
Factor V Leiden				
A	0,03	0,0225	0	0,0267
G	0,97	0,9775	1,0	0,9733
Prothrombin				
A	0,01	0,0074	0	0,0089
G	0,99	0,9926	1,0	0,9911
MTHFR				
T	0,28	0,3161	0,4583	0,2857
C	0,72	0,6839	0,5417	0,7143
TPA				
D	0,365	0,4705^a^	0,3636	0,5^b^
I	0,6350	0,5295	0,6364	0,5

^a^p =0,026; odds ratio (OR) 1,54; 95% confidence intervals (CIs) 0,99-2,4.

^b^p= 0,013; OR 1,74; 95% CIs 1,08-2,78.

**Table 6:** Genotype frequencies of four polymorphisms in ONFH patients in the relation to gender (total, female and male) and controls

**Genotype**	**Controls N=100**	**Patients**		
		**Total N=68**	**Female n=12**	**Male n=56**
Factor V Leiden				
GA	3(3%)	3(4,41%)	0	3(5,66%)
GG	97(97%)	65(95,59%)	12(100%)	53(94,34%)
Prothrombin				
GA	2(2%)	1(1,47%)	0	1(1,78%)
GG	98(98%)	67(98,53%)	12(100%)	55(98,22%)
MTHFR				
TT	7(7%)	4(5,88%)	2(18,66%)	2(3,57%)
CT	42(42%)	35(51,47%)	7(56,34%)	28(50,01%)
CC	51(51%)	29(42,65%)	3(25%)	26(46,42%)
TPA				
DD	18(18%)	13(19,12%)	2(16,66%)	11(19,64%)
ID	37(37%)	38(55,88%)^a^	4(33,34%)	34(60,72%)^b^
II	45(45%)	17(25%)	6(50,0%)	11(19,64%)

^a^p=0,004; odds ratio(OR ) 2,71; 95% confidence intervals (CIs) 1,32-5,57.

^b^p=0,0007; OR 3,75; 95% CIs 1,67-8,42.

**Table 7:** Allele frequencies of four polymorphisms in ONFH patients in the relation to age (total, <50 and >50) and controls

**Allele**	**Controls N=200**	**Patients**		
		**Total N=136**	**<50 n=86**	**>50 n=50**
Factor V Leiden				
A	0,03	0,0225	0,0117	0,041
G	0,97	0,9775	0,9883	0,959
Prothrombin				
A	0,01	0,0074	0,0117	0
G	0,99	0,9926	0,9883	1,0
MTHFR				
T	0,28	0,3161	0,267	0,38
C	0,72	0,6839	0,733	0,62
TPA				
D	0,365	0,4705^a^	0,4302	0,54^b^
I	0,6350	0,5295	0,5698	0,46

^a^p =0,026; odds ratio (OR) 1,54; 95% confidence intervals (CIs) 0,99-2,4.

^b^p= 0,018, OR= 2,04; 95% CIs 1,09-3,82.

**Table 8:** Genotype frequencies of four polymorphisms in ONFH patients in the relation to age (total, <50 and >50) and controls

**Genotype**	**Controls N=100**	**Patients**		
		**Total N=68**	**<50 n=43**	**>50 n=25**
Factor V Leiden				
GA	3(3%)	3(4,41%)	3(6,67%)	0
GG	97(97%)	65(95,59%)	40(93,33%)	25(100%)
Prothrombin				
GA	2(2%)	1(1,47%)	1 (2,38%)	0
GG	98(98%)	67(98,53%)	42(97,62%)	25(100%)
MTHFR				
TT	7(7%)	4(5,88%)	1(2,32%)	3(12%)
CT	42(42%)	35(51,47%)	22(51,16%)	13(52%)
CC	51(51%)	29(42,65%)	20(46.52%)	9(36%)
TPA				
DD	18(18%)	13(19,12%)	8(18,6%)	5(20%)
ID	37(37%)	38(55,88%)^a^	21(48.83%)	17(68%)^b^
II	45(45%)	17(25%)	14(32,57%)	3(12%)

^a^p=0,004; odds ratio(OR ) 2,71; 95% confidence intervals (CIs) 1,32-5,57.

^b^p=0,001; OR 6,89; 95% CIs 1,87-25,84.

**Table 9:** Allele frequencies of four polymorphisms in ONFH patients in the relation to number of affected joints (total, unilateral and bilateral ONFH) and controls

**Allele**	**Controls N=200**	**Patients**		
		**Total N=136**	**Unilateral n=58**	**Bilateral n=78**
Factor V Leiden				
A	0,03	0,0225	0,0334	0,0128
G	0,97	0,9775	0,9656	0,9872
Prothrombin				
A	0,01	0,0074	0,0175	0
G	0,99	0,9926	0,9825	1,0
MTHFR				
T	0,28	0,3161	0,3103	0,3205
C	0,72	0,6839	0,6897	0,6795
TPA				
D	0,365	0,4705^a^	0,3965	0,5256^b^
I	0,6350	0,5295	0,6035	0,4744

^a^p =0,026; odds ratio (OR) 1,54; 95% confidence intervals (CIs) 0,99-2,4.

^b^p= 0,01; OR= 1,92; 95% CIs 1,13-3,27.

**Table 10:** Genotype frequencies of four polymorphisms in ONFH patients in the relation to number of affected joints (total, unilateral and bilateral ONFH) and controls

**Genotype**	**Controls N=100**	**Patients**		
		**Total N=68**	**Unilateral n=29**	**Bilateral n=39**
Factor V Leiden				
GA	3(3%)	3(4,41%)	2(6,89%)	1(2,56%)
GG	97(97%)	65(95,59%)	27(93,11%)	38(97,44%)
Prothrombin				
GA	2(2%)	1(1,47%)	1(3,44%)	0
GG	98(98%)	67(98,53%)	28(96,56%)	39(100%)
MTHFR				
TT	7(7%)	4(5,88%)	2(6,89%)	2(5,12%)
CT	42(42%)	35(51,47%)	14(48,27%)	21(53,84%)
CC	51(51%)	29(42,65%)	13(44,84%)	16(41,04%)
TPA				
DD	18(18%)	13(19,12%)	3(10,34%)	10(25,64%)^a^
ID	37(37%)	38(55,88%)^b^	17(58,62%)	21(53,84%)^c^
II	45(45%)	17(25%)	9(31,04%)	8(20,52%)

^a^p=0,034; odds ratio (OR) 3,12; 95% confidence intervals (CIs) 1,06-9,18.

^b^p=0,004; OR 2,71; 95% CIs 1,32-5,57.

^c^p=0,009; OR 3,19; 95% CIs 1,26-8,03.

## Discussion

Factor V Leiden is a protein which accelerates prothrombin activation and thrombin generation. Factor V is inactivated by activated protein C (APC) by proteolysis. APC serves as a balancing anticoagulant mechanism. The G1691A point mutation in the factor V gene alerts the first cleavage site involved in the inactivation of factor V by APC. Mutated factor is less efficiently degraded by APC (APC resistance) which leads to thrombophilia [16]. Factor V Leiden constitutes the most frequent cause of inherited thrombophilia. The mean prevalence of the mutation in healthy Caucasians is from 4 to 5% [17]. Factor V Leiden has been established as an important and unequivocal risk factor for venous thrombosis [12]. An analysis of venous thromboembolism showed the presence of the mutation in approximately 20% of cases and an associated risk increased fivefold [12].

Glueck et al. [6] reported a 2,8% frequency of factor V Leiden mutation in group of patients with ONFH. Zalavras et al. [18] reported a high frequency of the mutation of factor V Leiden among patients with ON of the femoral head (18%) in comparison to reference group (4,6%). The prevalence of mutations was significantly increased both in primary and secondary ON patients. The authors emphasized that the patients and control group were Caucasians from the same geographic area, thus eliminating ethnicity as confounding factors in the prevalence of the mutations. Björkman et al. [19] reported a high frequency of factor V Leiden and prothrombin gene mutations among patients with ONFH (17%) in comparison with the control group of (13%). Gene mutations were significantly higher in patients with idiopathic ONFH (29%) than the control or secondary ONFH group. The study was performed in southern Sweden where the prevalence of factor V Leiden mutation is one of the highest in the world with the frequency of 10%. Chang et al. [20] observed neither factor V Leiden, nor prothrombin gene mutations both in patients with ON of the femoral head and reference group. We also have not revealed both an increased Factor V Leiden and prothrombin gene polymorphism in patients with ONFH.

Prothrombin is a precursor of thrombin. The G20210A prothrombin gene mutation causes higher levels of prothrombin which leads to increased generation of thrombin and thrombophilia [13]. Prothrombin gene mutation is present in 1 to 5% of Caucasians and is associated with venous thrombosis [21]. None of the previous reports have confirmed independent and significant association of prothrombin gene mutation with ON of the femoral head [6,20]. Zalavras et al. [18] reported higher incidence of prothrombin gene mutation in idiopathic ON in comparison to reference group (8,7% versus 2,6%).

Hyperhomocysteinemia has been identified as an independent risk factor for thromboembolic diseases [22]. Homocysteine causes damage of vascular endothelial cells, propagates vascular smooth cells and enhances coagulation activity on vascular walls [23]. 5,10-methylenetetrahydrofolate reductase (MTHFR) is an enzyme that plays a role in remethylation of homocysteine. The C677T (alanine to valine) polymorphism in MTHFR is reported to be involved in elevated plasma total homocysteine level [24]. Glueck et al. [6] reported a statistically significant relationship between the incidence of ONFH and MTHFR C677T polymorphism and plasma total homocysteine levels in a group of 36 patients. Zalavras et al. [25] reported on statistically significantly higher 677TT homozygous genotype in 66 patients with ON of the femoral head than in the reference group. The incidence of 677TT homozygous genotype was more statistically significant in patients with primary osteonecrosis. Asano et al. [26] reported a lack of relationship between MTHFR C677T polymorphism in 31 Japanese patients with secondary ON of the femoral head after renal transplantation. Chang et al. [20] reported that C677T polymorphism plays a role in pathogenesis of ON of the femoral head in the Korean population. In contrast Kim et al. [27] reported on MTHFR C677T polymorphism in 443 patients with ONFH and 273 reference group. The authors have not found any relationship between MTHFR polymorphism and the incidence of ONFH both in primary and secondary ON in Korean patients.

The results from our study do not support the data reported by Glueck et al. [6] and Zalvaras et al. [25] concluding that statistically significant relationship between ONFH and MTHFR C677T polymorphism is related to Caucasian subjects. Results similar to our study were presented by Séguin et al. [28] and Mehsen et al. [29] reporting on lack of correlation between the incidence of ONFH and MTHFR C677T polymorphism in Caucasian patients.

Tissue-type plasminogen activator is a major component of fibrinolytic system which converts plasminogen to the active enzyme plasmin in the presence of fibrin. Plasmin in turn, digests the fibrin clot, forming soluble fibrin degeneration products. Analysis of the TPA gene has revealed a common Alu insertion/deletion (I/D) polymorphism in intron 8 [30]. Jern et al. [31] have demonstrated that there is a strong correlation between genotype and in vivo release rate of TPA. Subjects with the DD phenotype were found to have significantly lower unstimulated release of TPA. All 3 genotypes showed a proportional increase in response to stimulation but the DD group showed the lowest net release. The hypothesis that TPA Alu I/D genotype may be associated with arterial or venous thromboembolism has been evaluated in several clinical studies with mixed results [32,33]. The TPA gene polymorphism was found to be related with increased myocardial infarction among Dutch patients [34]. The II genotype was associated with 2-fold increase of myocardial infarction compared with DD genotype. These findings have not been confirmed by other studies. Hooper et al. [35] reported on a significant increase in the frequency of the DD genotype in Afro American patients with venous thromboembolism compared with controls. To the best of our knowledge the correlation between TPA polymorphisms and ONFH has not been studied previously. In our study we revealed the significantly increased frequency of D allele and DD homozygosis in the group of patients with idiopathic ONFH in comparison with control group. DD genotype is associated with decreased release of TPA, thus on the basis of our results we conclude that TPA polymorphism is a risk factor of ONFH in Polish patients. We have also found the higher incidence of D allele and DD genotype in cases bilateral ONFH, thus it may be a reason for more severe course of the disease. The frequencies of D allele and ID heterozygous were statistically increased in males and patients older than 50 years and in bilateral ONFH. The relationship between PAI-1 polymorphism and ONFH has been evaluated in other studies. Glueck et al. [36] reported on higher incidence of 4G homozygosity of PAI-1 polymorphism in patients with ONFH (41%) in comparison with control subjects (20%). 61% of patients had higher plasminogen activator inhibitor activity versus reference group (5%). The authors concluded that heritable hypofibrinolysis may be a major etiologic factor of primary ONFH. These results were confirmed later by Glueck et al. [6] in another group of patients with ONFH. These results were later confirmed by Kim et al. [37] who found significant association between PAI-1 polymorphisms and ONFH.

## Conclusions

In conclusion, our results suggest that etiology of ONFH may be associated with abnormalities of hypofibrinolysis. We have not found any evidence that thrombophilia may be a risk factor of ONFH among Polish patients. Our results demonstrate the influence of ethnicity in differences of genetic risk factors of ONFH. Further studies with larger size samples and more detailed exploration of hypofibrinolysis markers are needed to support present findings.

## Competing interests

The authors declare that they have no competing interest.

## Authors’ contribution

JG conception, design, performance of the study and writing. MB conception and design, revision, and final appraisal of the manuscript for publication. TM data collection and writing. AK associated in writing the paper. All authors read and approved the final manuscript.

## Pre-publication history

The pre-publication history for this paper can be accessed here:

http://www.biomedcentral.com/1471-2474/14/264/prepub
